# Osteoinductive Moldable and Curable Bone Substitutes Based on Collagen, BMP-2 and Highly Porous Polylactide Granules, or a Mix of HAP/β-TCP

**DOI:** 10.3390/polym13223974

**Published:** 2021-11-17

**Authors:** Andrey Vyacheslavovich Vasilyev, Valeriya Sergeevna Kuznetsova, Tatyana Borisovna Bukharova, Egor Olegovich Osidak, Timofei Evgenevich Grigoriev, Yuriy Dmitrievich Zagoskin, Irina Alekseevna Nedorubova, Sergey Petrovich Domogatsky, Igor Ivanovich Babichenko, Oksana Aleksandrovna Zorina, Sergey Ivanovich Kutsev, Sergei Nicolaevich Chvalun, Anatoly Alekseevich Kulakov, Fedor Fedorovich Losev, Dmitry Vadimovich Goldshtein

**Affiliations:** 1 Research Centre for Medical Genetics, Moskvorechye st., 1, 115478 Moscow, Russia; tilia7@yandex.ru (V.S.K.); bukharova-rmt@yandex.ru (T.B.B.); irina0140@gmail.com (I.A.N.); mgnc@med-gen.ru (S.I.K.); dv@rm7.ru (D.V.G.); 2 Central Research Institute of Dental and Maxillofacial Surgery, Timur Frunze st., 16, 119021 Moscow, Russia; babichenko-ii@rudn.ru (I.I.B.); zorina-cniis@yandex.ru (O.A.Z.); kulakov@cniis.ru (A.A.K.); losev@cniis.ru (F.F.L.); 3 Imtek Ltd., 3rd Cherepkovskaya st., 15a, 121552 Moscow, Russia; eosidak@gmail.com (E.O.O.); spdomo@yandex.ru (S.P.D.); 4 Federal State Budgetary Institution National Medical Research Center of Cardiology Ministry of Health of the Russian Federation, 3rd Cherepkovskaya st., 15a, 121552 Moscow, Russia; 5 NRC “Kurchatov Institute”, 1, Akademika Kurchatova pl, 123182 Moscow, Russia; timgrigo@yandex.ru (T.E.G.); zagos@inbox.ru (Y.D.Z.); chvalun_SN@nrcki.ru (S.N.C.); 6 Department of Pathological Anatomy, Peoples’ Friendship University of Russia (RUDN University), 6 Miklukho-Maklaya st., 117198 Moscow, Russia; 7 I.M. Sechenov First Moscow State Medical University of the Ministry of Health of the Russian Federation (Sechenov University), 119991 Moscow, Russia; 8 Moscow Institute of Physics and Technology (National Research University), 9 Institutskiy per., Dolgoprudny, 141701 Moscow, Russia

**Keywords:** collagen, fibronectin, hydrogel, polylactide, HAP/β-TCP, BMP-2, osteoinduction

## Abstract

In dentistry, maxillofacial surgery, traumatology, and orthopedics, there is a need to use osteoplastic materials that have not only osteoinductive and osteoconductive properties but are also convenient for use. In the study, compositions based on collagen hydrogel were developed. Polylactide granules (PLA) or a traditional bone graft, a mixture of hydroxyapatite and β-tricalcium phosphate (HAP/β-TCP), were used for gel filling to improve mechanical osteoconductive properties of compositions. The mechanical tests showed that collagen hydrogels filled with 12 wt% highly porous PLA granules (elastic modulus 373 ± 55 kPa) or 35 wt% HAP/β-TCP granules (elastic modulus 451 ± 32 kPa) had optimal manipulative properties. All composite components were cytocompatible. The cell’s viability was above 90%, and the components’ structure facilitated the cell’s surface adhesion. The bone morphogenetic protein-2 (BMP-2) provided osteoinductive composition properties. It was impregnated directly into the collagen hydrogel with the addition of fibronectin or inside porous PLA granules. The implantation of a collagen hydrogel with BMP-2 and PLA granules into a critical-size calvarial defect in rats led to the formation of the most significant volume of bone tissue: 61 ± 15%. It was almost 2.5 times more than in the groups where a collagen-fibronectin hydrogel with a mixture of HAP/β-TCP (25 ± 7%) or a fibronectin-free composition with porous PLA granules impregnated with BMP-2 (23 ± 8%) were used. Subcutaneous implantation of the compositions also showed their high biocompatibility and osteogenic potential in the absence of a bone environment. Thus, the collagen-fibronectin hydrogel with BMP-2 and PLA granules has optimal biocompatibility, osteogenic, and manipulative properties.

## 1. Introduction

The rise in musculoskeletal disorders and demand for dental bone grafts has led to an increased number of bone grafts used. In 2020, the market value for bone grafts was estimated to be worth approximately USD 2.65 billion and is projected to grow to approximately USD 3.36 billion by 2028, with a combined annual growth rate of 4.3% [1]. Autologous bone is still the current “gold standard” for bone defect repair [2]. It is non-immunogenic and possesses the essential components to achieve osteoinduction and osteoconduction. However, autografts require a secondary surgical procedure at the site of the tissue harvest that is associated with high surgical risks, such as bleeding, inflammation, infection, chronic pain, and higher costs [3].

In craniomaxillofacial surgery, autografts play a marginal role because allografts widely overtake them for reasons related to costs/benefits [4]. Allografts account for more than 50% of the market for bone grafts [1]. They are effective and safe bone grafts with osteoconductive properties. However, their processing and sterilization result in the loss of osteogenic capacity [5].

To overcome previously described disadvantages of bone grafts, bioactive bone materials have been developed [6]. They consist of a carrier and an osteogenic or osteoinductive component (cells, proteins, or genetic constructs). One of the more popular bioactive materials is the Infuse Bone Graft. It is a collagen sponge wetted with a BMP-2 solution. This material is highly effective for bone regeneration in traumatology and orthopedics. However, it also has disadvantages: it requires additional reinforcement constructs or cages for extensive bone defect restoration, and the used supraphysiological concentration of BMP-2 can cause adverse effects [7].

Bone cement was one of the first synthetic materials developed for replacing significant bone defects. Its mechanical properties allow extended bone defects’ restoration, and the ability of hardening makes it convenient for use [8]. However, its low resorption rate and solid structure limit vascular ingrowth and cell penetration, reducing the bone formation rate.

Thus, bone grafts for dentistry, traumatology, and orthopedics must have several properties combined. They need to have osteoinductive and osteoconductive properties and be biocompatible and resorbable. Additionally, bone grafts should be convenient and maintain a given shape when replacing complex bone defects. Unfortunately, there is no such material on the market. In this regard, the development of bone graft with the described properties is required, and in the future, it will form a new generation of osteoplastic materials.

Previously, we carried out several successful studies on preparing thermosetting biocompatible materials based on chitosan hydrogels and polylactide (PLA) fillers with BMP-2 [9,10]. The materials showed the ability to cure at the body temperature, high biocompatibility and resorbability, and demonstrated marked osteoinductive and osteoconductive properties in vitro and in vivo. However, difficulties in obtaining chitosan with reproducible properties forced us to pay attention to a more traditional tissue engineering biopolymer-collagen.

Type I collagen is a natural component of bone tissue, and its degradation products can be involved in synthesizing a new bone matrix [11]. However, collagen hydrogels have insufficient mechanical and osteoinductive properties and require the inclusion of fillers and growth factors. As we have shown earlier, highly porous polylactide granules could be a good filler. They can release small doses of BMP-2 for a long time, provide ecto- and orthotopic neo-ostegenesis, and exclude possible complications associated with an excessive concentration of BMP-2: hyperostosis, inflammation, and anaplasia [12,13].

As an alternative filler, we chose material Indost (NPK Polystom, Russia), containing hydroxyapatite (HAP) and β-tricalcium phosphate (β-TCP). As well as collagen, HAP and β-tricalcium phosphate are bone constituents and can be used for bone formation [14]. Despite the excellent biological properties of collagen hydrogel and mix HAP/β-TCP, they do not allow the prolonged release of BMP-2. Fibronectin was used to promote the slow release of BMP-2 from the collagen hydrogel with HAP/β-TCP filling. Its competitive adhesion leads to the protein’s slow release from the collagen–BMP-2 complex [12,15].

This study aimed to develop osteoplastic compositions based on collagen hydrogel, fibronectin, BMP-2, highly porous polylactide granules, and a mixture of HAP and β-tricalcium phosphate granules, which would have optimal mechanical and biological properties and would be convenient for use.

## 2. Materials and Methods

### 2.1. Components’ Fabrication

#### 2.1.1. Highly Porous Polylactide Granules’ Fabrication

PLA granules’ fabrication was performed following a previously developed method [16]. Polylactide emulsions (NatureWorks© LLC, Minnetonka, MN, USA Ingeo 4032) were dissolved in 1,4-dioxane. Granules were prepared by spraying and freeze-drying. They had a 0.1–0.2 mm diameter and 98% porosity. Gamma irradiation of 15 kGy was used for sterilization.

#### 2.1.2. Collagen-Fibronectin Hydrogel Fabrication

A sterile 10% neutral solution of porcine atelocollagen type I (Viscoll^®^, cat.PA100, Imtek Ltd., Moscow, Russia) was mixed with human fibronectin (cat. H Fne-C, Imtek Ltd., Moscow, Russia) in a ratio of 1: 4 by volume. The resulting mixture was incubated at 4 °C for 24 h.

#### 2.1.3. BMP-2 Impregnation

A stock solution with 10 μg BMP-2 was used. Recombinant human BMP-2 (rhBMP-2, AkronBiotech, Boca Raton, FL, USA, SKU: AK8356, obtained in E. Coli) was dissolved in a buffer containing BSA and 10 mM acetic acid. The PLA and HAP/β-TCP granules were wetted by BMP-2 solution. After, PLA granules were vacuumed five times. Subsequent freeze-drying led to protein adsorption on the surface and in the pores of polylactide granules. Collagen-based hydrogels were mixed with the BMP-2 solution to obtain compositions with osteoinductive properties.

#### 2.1.4. Fabrication of Composite Materials

Polylactide granules were chosen as fillers for collagen hydrogel. They were added with constant stirring of the gel for uniform distribution in the volume until a mass fraction of 4, 8, 12, and 16% was reached.

The collagen-fibronectin hydrogel was mixed with HAP/β-TCP (Indost, NPK Polystom, Moscow, Russia) to obtain compositions with a mass fraction of granules of 10, 20, 35, and 50%.

### 2.2. Investigation of Physical and Mechanical Properties

Rheological measurements were conducted in an Anton Paar Physica MCR 501 rheometer (Anton Paar GmbH, Graz, Austria) with a cone-and-plate measuring system CP50. Measurements were performed in an amplitude scan mode at a frequency of 10 rad/s.

The mechanical strength of the composite material was analyzed by measuring Young’s Modulus at room temperature using a Universal Testing Machine (UTM) (Instron 5965, Instron Corp., Norwood, MA, USA). The deformation speed was 50%/min with a 10 mm width and ~10 mm length. Compression tests were performed using a universal testing machine (Instron 5965, Instron Corp., Norwood, MA, USA).

The developed compositions were visually and tactilely analyzed by a group of dentists.

### 2.3. In Vitro Stvitroudies

#### 2.3.1. Cell Viability

Collagen-fibronectin and collagen hydrogels and PLA and HAP/β-TCP granules were studied in vitro. The materials were added to a 24-well plate, along with cell culture media (DMEM (PanEco, Moscow, Russia) with 10% FBS (Gibco; Thermo Fisher Scientific, Inc., Waltham, MA, USA), 2 mM L-glutamine (PanEko, Moscow, Russia), and 100 mg amikacin (Sintez, Kurgan, Russia), and they were incubated for seven days under standard culture conditions (at 37 °C and 5% CO_2_).

For testing, cultures of human adipose tissue mesenchymal stem cells (AT MSC) and stem cells from human exfoliated deciduous teeth (SHED) at 3–4 passages were used. Cells were detached from the surface of culturing Petri dishes using a Versene solution (PanEco, Moscow, Russia) with the addition of 0.25% trypsin (PanEco, Moscow, Russia) and seeded in 96-well plates (Nunc, Thermo Fisher Scientific, Inc., Waltham, MA, USA) at a density of 5000–10,000 cells per well. After culturing for one day, the medium incubated with the samples was added to the wells. The cells were cultured for 1 and 7 days. The control group was supplemented with a culture medium. Then, a solution of MTT (tetrazolium (3-(4,5-dimethyl thiazolyl-2)-2,5-diphenyltetrazolium bromide); PanEco, Moscow, Russia) at a concentration of 0.5 mg/mL was added to the wells and incubated for 2 h at 37 °C. Formazan crystals were extracted from cells using dimethylsulfoxide (DMSO, PanEco, Moscow, Russia). The absorption of formazan was assessed by measuring the optical density of the eluate at a wavelength of 570 nm on an EnSpire Multimode Plate Reader (PerkinElmer, Waltham, MA, USA).

#### 2.3.2. Study of Cell Adhesion

Cell adhesion was investigated using a fluorescent dye. For this, a cell suspension was prepared. MSC cells were removed from Petri dishes with a Versen solution (PanEko, Moscow, Russia) with 0.25% trypsin (PanEko, Moscow, Russia) and centrifuged at 1100 rpm for 10 min. Cells were stained with PKH-26 dye according to the manufacturer’s instructions (Sigma-Aldrich, St. Louis, MO, USA). After staining, the cells were precipitated by centrifugation. The concentration of cells in suspension was 1 × 10^6^ per 1 mL of growth medium. The samples of materials were placed in 96-well plates (Thermo Fisher Scientific, Inc., Waltham, MA, USA). A total of 30 μL of the cell suspension in each well were added and incubated for 60 min under standard culture conditions. After, 1 mL of growth medium was carefully added. The cells were cultured on carriers for 7 days, with the growth medium changing every 3–4 days. Images of cells labeled with PKH26 on the material’s surface were obtained using an AxioObserver D1 inverted fluorescence microscope with an AxioCam HRc camera (Carl Zeiss, Oberkochen, Germany).

### 2.4. In Vivo Studies

The collagen-fibronectin gel containing BMP-2, collagen-fibronectin gel with BMP-2 and HAP/β-TCP granules, collagen-fibronectin gel with BMP-2 and polylactide granules, and collagen hydrogel with polylactide granules impregnated with rhBMP-2 were studied in vivo. Sixty male Wistar rats weighing 300–350 g were used in this experiment. All rats were randomly divided into six groups depending on the type of the material and implantation site. There were 12 rats in the critical-size calvarial defect implantation groups and 6 in the subcutaneous implantation groups.

All protocols in this study were approved by the Committee on the Ethics of Animal Experiments of the Central Research Institute of Dentistry and Maxillo-facial Surgery, Moscow (IACUC permit number: 2019-293), in compliance with the Guide for the Care and Use of Laboratory Animals published by the US National Institutes of Health (NIH publication no. 85–23, revised 1996), European Convention for the Protection of Vertebrate Animals used for Experimental and Other Scientific Purposes, and ISO 10993–22006. Rats were intraperitoneally anesthetized with 30 mg/kg Zoletil (Virbac SA, Carros, France) and 5 mg/kg Xylazine (Interchemie Werken “De Adelaar” BV, Venray, The Netherlands).

#### 2.4.1. Orthotopic Osteogenesis Model: Implantation into a Rat Critical-SIZE Calvarial Defect

Operations were performed following the previously developed method [17]. After shaving and disinfection, transverse and vertical laterally displaced incisions of the scalp were made, forming a triangular flap. The periosteum was sharply divided and then gently pushed laterally. A full-thickness calvarial bone defect was created using a C-reamer trepan of 5.5 mm diameter and 1.5 mm height from the SLA Kit (Neobiotech, Seoul, Korea) with sterile regular saline irrigation. After implantation, the periosteum and skin were sutured with Vicryl 5/0 (Ethicon, NJ, USA).

#### 2.4.2. Ectopic Osteogenesis Model: Subcutaneous Implantation in Rats

Subcutaneous implantation in rats was performed as selected in the previous step compositions. This model helps to reveal the osteoinductive properties of the compositions in a region without a bone environment and confirms the composition’s efficacy for bone regeneration. After shaving and ethanol disinfection of skin, one dorsum midline incision was made. Two pockets on each side of the incision were formed. Materials were implanted into subcutaneous pockets, and the wounds were sutured with Vicryl 5/0 (Ethicon, Raritan, NJ, USA).

#### 2.4.3. Euthanasia, Histological Examination, and Morphometry

Four weeks after surgery, all rats were euthanized by CO_2_ inhalation. The implantation sites were resected and fixed in 10% neutral-buffered formalin for 24–48 h. Then, they were washed in running water for 24 h, decalcified in 20% EDTA for five weeks, dehydrated in alcoholic solutions, and embedded in paraffin wax. The thickness of the tissue sections was 5–7 μm. Slides were stained with hematoxylin-eosin and Masson trichrome (with aniline blue) (Biovitrum, St. Petersburg, Russia). The slides were scanned on an Axioimager M.1 light microscope (Carl Zeiss, Oberkochen, Germany) using the Zen Pro 3.0 software complex. Subsequently, the acquired images were used for histological examination and osteogenesis character determination. Additionally, they were used to subject the quantitative analysis to new bone volume using Adobe Photoshop CS5 software regarding the bone volume over total material volume. The morphometric analysis was performed on the six serial sections for each sample, according to the generally accepted recommendations [18,19].

### 2.5. Statistical Analysis

Statistical analysis and graphing were performed with GraphPad Prism software (GraphPad Inc, La Jolla, CA, USA). Intergroup differences were statistically analyzed by a Student’s t-test. Differences were considered significant when *p* < 0.05. When constructing bar graphs, the arithmetic mean and standard deviation were depicted. The strength of differences was designated in accordance with the requirements of the American Physiological Association (APA): “*” corresponds to *p* < 0.05; “**” corresponds to *p* < 0.01; “***” corresponds to *p* < 0.001. The data are presented as average ± SD.

## 3. Results

### 3.1. Mechanical Properties

#### 3.1.1. Collagen Hydrogels

**Figure 1:**
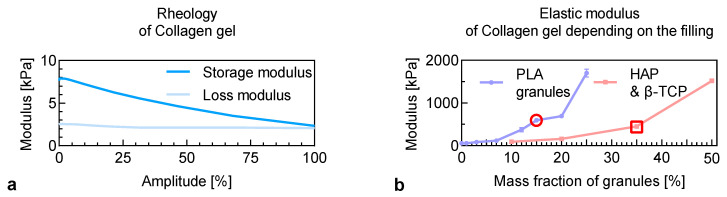
Rheological (**a**) and mechanical properties (**b**) of collagen-fibronectin hydrogel after curing and using various fillers.

Fibronectin was used in small amounts for obtaining the collagen-fibronectin hydrogel. According to visual and manipulative tests data, the mechanical properties of these hydrogels were similar to the collagen hydrogel. After curing at body temperature, collagen and collagen-fibronectin hydrogels obtained elastic properties. An accumulation module exceeded the loss module in the entire amplitude range (Figure 1a). A linear decrease in the difference between these modules was observed over the entire range.

However, the hydrogels could not keep their shape before curing. In this regard, a mixture of HAP/β-TCP granules or highly porous PLA granules was used as the fillers.

#### 3.1.2. Hydrogel Filled with a Mixture of HAP/β-TCP

A mixture of HAP with β-TCP granules was chosen as a filler for collagen hydrogels. The modulus of elasticity of hydrogel filling with HAP/β-TCP granules significantly increased with their number (Figure 1b). The visual and manipulative tests showed that filling a hydrogel with more than 35 wt% violated the granules’ adhesion to the matrix. The material filled with 50 wt% granules was crumbled in the hand when modeling various geometric shapes. For this reason, for further research, a composition with a 35 wt% HAP/β-TCP was chosen.

#### 3.1.3. Hydrogel Filled with Highly Porous PLA Granules

The results show that polylactide granules increased the compositions’ elastic modulus from 80 kPa to 400–2700 kPa in a hydrogel filled with 12–20 wt% PLA granules and 1.8 MPa with 25 wt%. Despite the increase in elastic modulus values, the limit deformation was reduced. It was connected with incomplete wetting of particles with the hydrogel phase and the materials’ embrittlement. The elastic modulus of materials containing 12 and 15 wt% PLA granules was not significantly different. Compositions with a PLA of less than 10 wt% showed a low modulus of elasticity. They could not take the necessary geometrical shape before curing. Based on mechanical data and convenience of use, a system with 12 wt% of the particle was chosen for further studies.

#### 3.1.4. Comparison of Fillers’ Mechanical Properties

**Figure 2:**
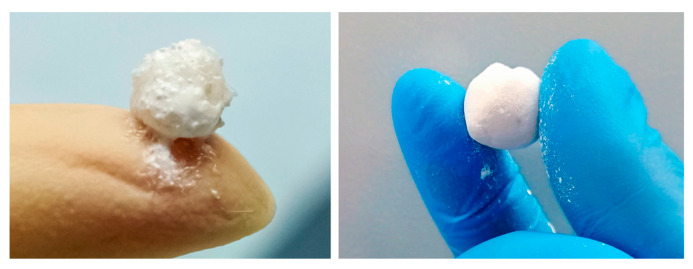
Hydrogels filled with a mixture of HAP and β-TCP (**left**) and porous PLA granules (**right**) rolled into a ball.

When comparing systems with the maximum filling before the material destruction, a highly porous PLA granules composition showed higher stress than a system with HAP/β-TCP granules. However, the filling of hydrogels with porous PLA granules increased their elastic modulus while maintaining elasticity. Additionally, the better moldable properties of composition with PLA granules were confirmed by visual and manipulative analysis. The composition did not stick to gloves. Rolled into a ball, it had smoother edges than the composition filled with HAP/β-TCP granules (Figure 2).

### 3.2. In Vitro Research

#### 3.2.1. Collagen and Collagen-Fibronectin Gels

**Figure 3:**
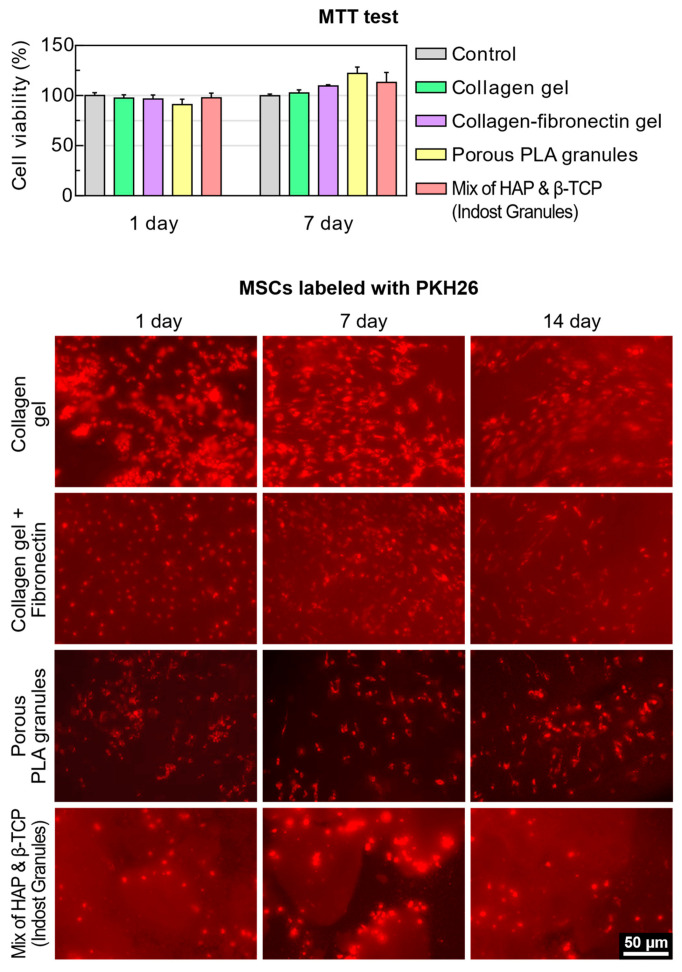
Cell viability and cell adhesion on the materials’ components.

The highly purified collagen and collagen-fibronectin hydrogels showed high bio-compatibility in the MTT test. There were no statistically significant changes in the relative viability of cells after 1, 4, and 7 days of incubation in the presence of collagen and collagen-fibronectin hydrogels. The addition of fibronectin to the collagen gel did not provoke cell death. At the observation period, collagen hydrogel and collagen-fibronectin hydrogel promoted cell adhesion (Figure 3).

#### 3.2.2. The Mixture of HAP Granules with β-TCP

The results of the MTT test showed that a mixture of HAP with β-TCP did not have a cytotoxic effect on cells. By the end of the seventh day, a statistically significant increase in the relative viability of cells was noted. In addition, the porous structure of the granules promoted cell adhesion. From the first day of the experiment, the cells were evenly distributed over the surface of the granules, changing their shape from polygonal to elongated fusiform by 7 days. However, in some areas, heterogeneity in the structure of the granules resulted in cells being rolled (Figure 3).

#### 3.2.3. Highly Porous PLA Granules

Highly porous PLA granules showed high cytocompatibility. By the end of the seventh day, they led to an increase in the relative survival of cells. According to fluorescence microscopy data, the porous structure promoted the penetration of cells into the granules, their spreading, and their assuming an elongated or polygonal shape (Figure 3).

### 3.3. In Vivo Study

#### 3.3.1. Orthotopic Osteogenesis Model

##### Collagen Fibronectin Gel with BMP-2

**Figure 4:**
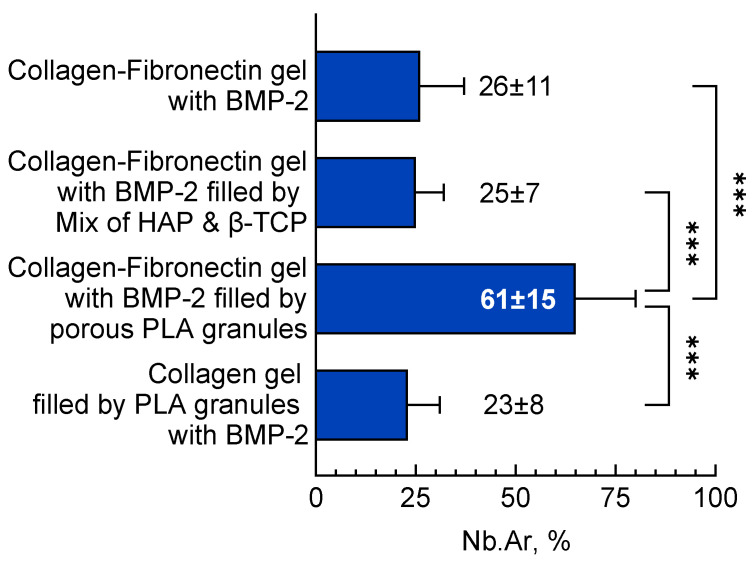
Newly formed bone tissue volume after implantation of different compositions into rat critical-size calvarial defects. ***– *p* < 0.001.

**Figure 5:**
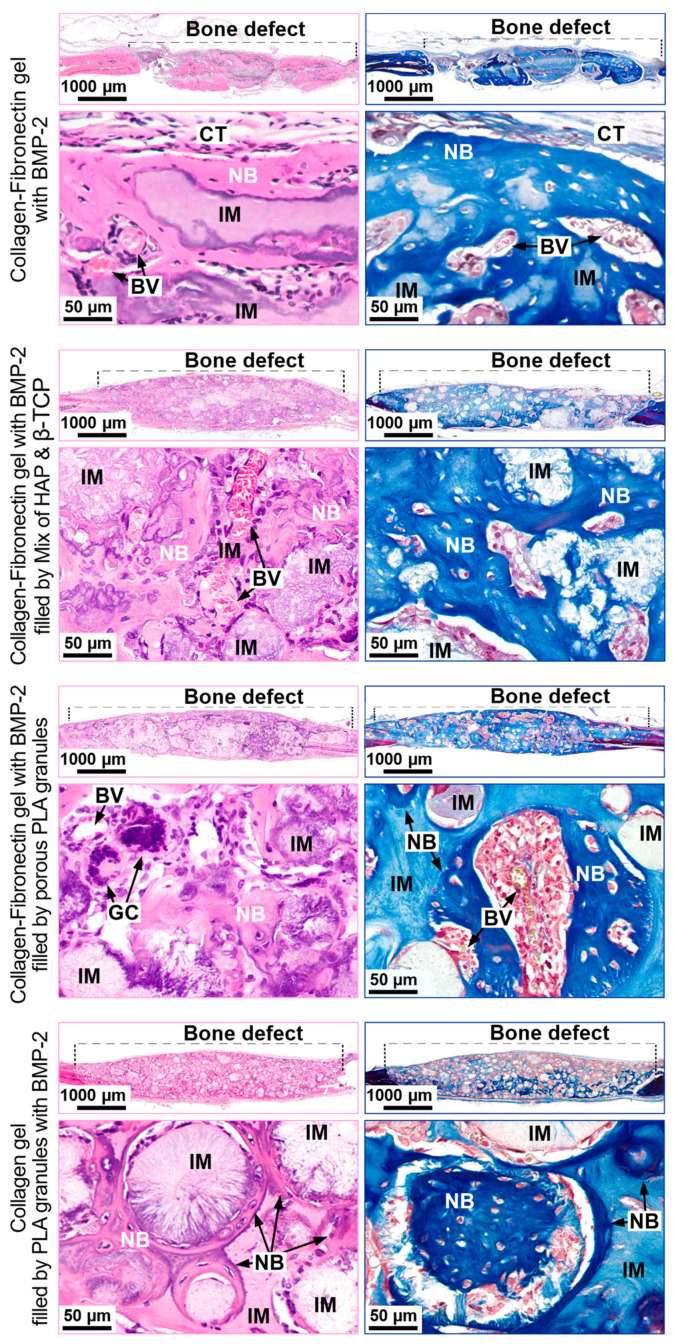
Implantation of materials into rat critical-size calvarial defect. H&E (**left** column) and Masson (**right** column) staining. IM—Implanted material, CT—connective tissue, BV—blood vessel, NB—newly formed bone, GC—giant cells.

After 28 days in the implantation area, bone formation was observed from the periphery of the material to its center. According to morphometric analysis, the volume of newly formed tissue was 26 ± 11% (Figure 4). In rare fields of vision, single-plasma cells and lymphocytes leaving vessels were detected. This indicated the absence of a severe inflammatory response. (Figure 5).

##### Collagen-Fibronectin Gel with BMP-2 Filled with HAP/β-TCP Granules

**Figure 6:**
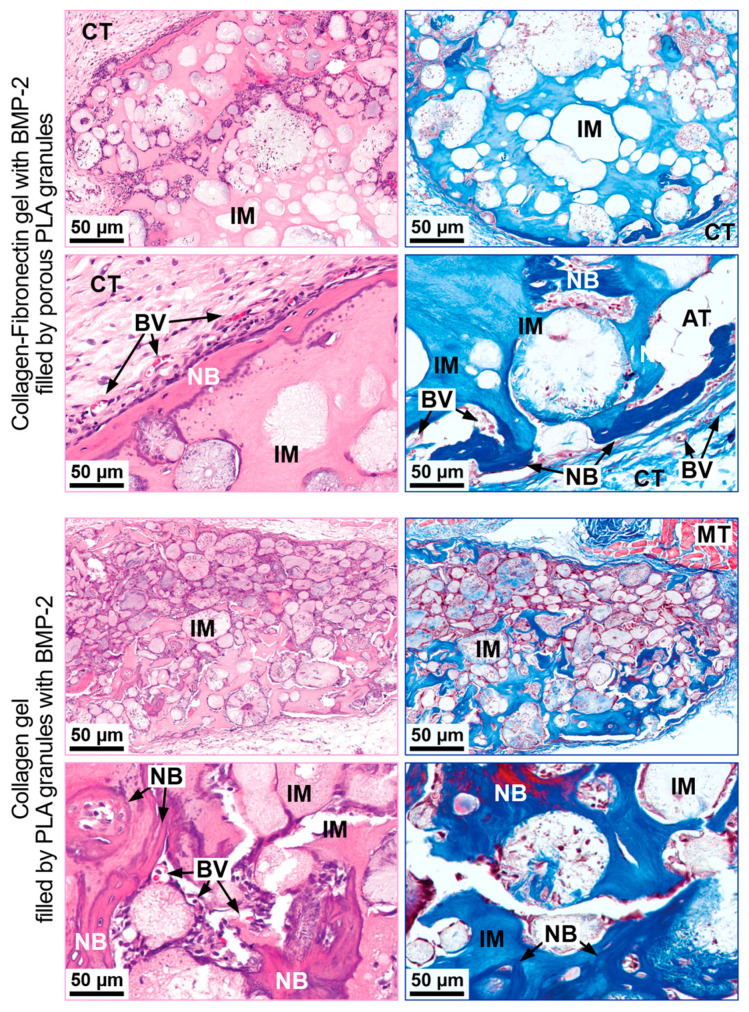
Subcutaneous implantation of the materials. H&E (**left** column) and Masson staining (**right** column). IM—implanted material, CT—connective tissue, BV—blood vessel NB—newly formed bone, GC—giant cells, AT—adipose tissue, MT—muscle tissue.

After 28 days, bone formed both in the thickness of the material and along its periphery (Figure 5). At the sites of vascular ingrowth, the collagen-fibronectin hydrogel was replaced by young lamellar bone tissue. Granules of HAP and β-TCP were surrounded by foreign-body giant cells. Additionally, single plasma cells and lymphocytes leaving vessels were identified. These results suggest the absence of a marked inflammatory response. The volume of newly formed bone tissue was 25 ± 7% (Figure 6).

##### Collagen-Fibronectin Gel with BMP-2 Filled with PLA Granules

The formation of a bone tissue “crust” along the border of the implanted material was observed (Figure 5). The polylactide granules were surrounded by foreign-body giant cells. The blood vessels grew into the thickness of the material. Foci of new bone tissue were found around the blood vessels. The newly formed bone tissue volume was the largest between experimental groups: 61 ± 15% (Figure 4).

##### Collagen Gel with PLA Granules Impregnated with BMP-2

In the composition containing polylactide granules impregnated with BMP-2 and fibronectin was not used. The osteoinductive protein was associated with PLA, so there was no need to displace it from the collagen hydrogel. After 28 days in the implantation area, foci of new bone grew around PLA granules (Figure 5). The volume of the new bone tissue was 23 ± 8% (Figure 4).

#### 3.3.2. Ectopic Osteogenesis Model

Implantation into the critical-size calvarial defect showed that the most significant bone formation was observed in a group with a collagen-fibronectin gel with BMP-2 filled with polylactide granules. The composition subcutaneous implantation was performed to confirm the osteogenic properties. A collagen hydrogel with polylactide granules impregnated with BMP-2 was used as a comparison group.

##### Collagen-Fibronectin Gel with BMP-2 Filled with PLA Granules

Foci of neo-osteogenesis were observed along the periphery of the gel boundaries. As in the case of critical-size calvarial defect implantation, the newly formed lamellar bone tissue formed a “crust” on the surface of the material (Figure 6). At the same time, new bone tissue areas in the thickness of the material were rarely observed. This indicates that the formation of bone tissue along the periphery could serve as a barrier preventing the resorption of the composition and the ingrowth of vessels into the material.

##### Collagen Gel with PLA Granules Impregnated with BMP-2

The foci of osteogenesis in the implantation area had a young lamellar bone tissue structure. They surrounded the PLA granules and grew into their thickness with appositional growth centers (Figure 6). In places of polylactide granules concentration, more extensive osteogenesis occurred. The collagen gel was replaced by connective tissue, and foreign-body giant cells surrounded the highly porous polylactide granules.

## 4. Discussion

The results of this study showed that the developed materials had promising properties for bone tissue regeneration. However, their mechanical and biological properties were different depending on the composition of the materials. The results of the mechanical properties study showed that the composition with PLA granules had better mechanical properties than the composition with HAP/ β-TCP granules. Its elastic modulus was 373 ± 55 kPa, whereas for HAP/β-TCP, it was 451 ± 32 kPa. This was related to the high porosity (98%) of PLA granules that provided linkage formation between the hydrogel and granules. PLA granules increased composition viscosity and prevented its destruction with increasing elastic deformation. Additionally, a blending of hydrogel with PLA granules was accompanied by their crumbling. This made it more possible to obtain a homogenous structure and smooth surface without sharp, protruding elements than in a material containing a mixture of HAP and β-TCP. As for osteoinductive properties, the results showed that the material filled with PLA granules induced the formation of 61 ± 15% new bone, whereas in the composition with HAP/β-TCP, the volume was 25 ± 7%. This means that PLA granules were a preferable filler for collagen-fibronectin hydrogels than the mixture of HAP and β-TCP, both for mechanical properties’ improvement and protein carrying. Our previous study showed that implantation of HAP with low concentrations of BMP-2 did not induce heterotopic osteogenesis, while PLA granules with rhBMP-2 provided new bone formation. [16]. In our view, this was connected with the retention of BMP-2 on the HAP surface. These effects related to an obstacle of HAP to the action of BMP-2, as shown in other studies [20,21,22]. When comparing the collagen-fibronectin hydrogel and PLA granules for BMP-2 delivery, we found that hydrogel is a more effective carrier for osteogenetic protein. The collagen-fibronectin hydrogel with BMP-2 formed new bone tissue in a volume 2.6 times greater than the composition with BMP-2 impregnated in PLA granules. In our view, this could be related to features of impregnation technology and release kinetics of BMP-2. As shown previously, BMP-2 losses in the collagen-fibronectin hydrogel during protein release was 11% less than in the PLA granules [12]. Additionally, hydrogel had a larger contact area with the surrounding tissues than PLA granules. As a result, released from gel, BMP-2 promoted the differentiation of more cells, resulting in more effective new bone formation. Thus, the composition of the collagen-fibronectin hydrogel with BMP-2 and PLA granules showed optimal properties compared to other developed materials. It expressed elastic properties, homogenous structure, and convenience in use. Additionally, it showed biocompatibility and osteoconductive and osteoinductive properties and resulted in bone formation in orthotopic and ectopic osteogenesis models.

These results allow us to compare developed compositions with different types of bone graft materials. According to studies, implantation of autogenous bone chips into a rat critical-sized calvarial defect leads to the formation of a small bone volume: 8.9 ± 4.3% [23] or 6.6 ± 1.8% [24]. In our experiment, the implantation of the compositions led to 23 ± 8% to 61 ± 15% new bone volume. The osteogenic properties of the developed compositions are also superior to those of traditional bone graft materials associated with human bone cells, impregnated with an osteoinductive protein or mixed with autogenous bone tissue. The bone grafting with BCP granules associated with human bone cells led to a 6.6 ± 2.8% new bone volume density. This result could be related to insufficient osteogenicity of cells due to poor blood supply which caused cells death. Upon implantation of Bio-Oss granules moistened with BMP-2 into a critical-size calvarial defect of rats, the volume of newly formed bone tissue was 2.6 ± 1.1% [16]. Implantation of a mixture of autogenous bone tissue with Bio-Oss in a ratio of 50/50 or 25/75 in the critical-size calvarial defects of rabbits was accompanied by the formation of 3–3.5% newly formed bone [25]. The unstable interaction between bone graft materials and substances induced bone formation resulted in non-prolonged prolonged release kinetics and a significant decrease in their osteoinductive effectiveness [26].

The osteogenic properties of the existing bioactive bone grafts analogs are contradictory and require attention. The most common and well-known commercial BMP-2-containing material is the Infuse Bone Graft. In one study, the Infuse Bone Graft implantation, after four weeks, resulted in the volume of newly formed bone tissue, which, compared to the total tissue volume, was about 9% [26]. This result could be related to the difference in the animal model. In the described study, mice were used, while we used rats. In another study in rats, Infuse Bone Graft stimulated bone formation in a volume of 99.4 ± 1.8% [27]. Such a result could be related to the morphometrical measurement technique. The data were obtained from X-ray and histological examination and described the filling of the defect with bone in general but not the percentage of newly formed bone tissue. In addition, upon careful analysis of the presented histological micrographs, it can be assumed that the authors calculated the volume of the regenerate relative to the volume of the original defect. The excess growth of bone tissue outside the defect was also considered, leading to overestimating the results, bringing them closer to 100%.

The BMP-2 dosage in the Infuse Bone Graft deserves special attention. According to studies, it is supraphysiological, and a concentration of 1.5 mg/mL is 150 times higher than in the developed compositions. Several studies have shown that high doses of BMP-2 in the Infuse Bone Graft lead to complications such as hyperostosis, inflammation, and anaplasia [28]. Additionally, using the clinical dose of BMP-2 in one study resulted in less bone volume formation and caused ectopic bone growth compared with the groups with a lower BMP-2 concentration [26]. In our view, a high concentration of BMP-2 in Infuse Bone Graft is related to the form of carrier. The collagen sponge cannot provide slow release of BMP-2, maintain long-term protein activity, and, as a result, stimulate effective osteogenesis with a low BMP-2 dose. As for the developed compositions, the previous study showed that a collagen-fibronectin hydrogel and PLA granules can prolong the release of BMP-2 at the minimum effective dosage [12]. In addition, the collagen sponge used as a carrier for BMP-2 is inconvenient for the grafting of bone defects. After wetting with BMP-2 solution, the sponge loses its original volume and cannot be used to restore extended defects or those having a complex anatomical shape without additional reinforcing structures. Additionally, the Infuse Bone Graft implantation can be accompanied by BMP-2 leakage into the surrounding tissues [28]. The plasticity and thermosetting allow the developed materials to have excellent convenience of in-use properties and preservation of the impregnated BMP-2 volume. They could be used for complex shape defect restorations without using additional structures. These properties provide the advantage of the developed compositions over autogenous bone tissue and most other bone grafts.

## 5. Conclusions

This study successfully prepared compositions based on collagen hydrogel, fibronectin, polylactide granules, and rhBMP-2 for bone tissue regeneration. The elasticity and thermosetting properties made the materials convenient for use. The cell’s viability was above 90%, indicating cytocompatibility of the components. The osteoinductive properties of the materials allowed inducing the formation of new bone, both in subcutaneous implantation and in critical-size calvarial defects, in volumes up to 61 ± 15%. The developed compositions of thermosetting material based on collagen hydrogel, fibronectin, polylactide granules, and rhBMP-2 were studied. The collagen-fibronectin hydrogel with BMP-2 and PLA granules showed promising biological, mechanical, and manipulative properties. Despite the obtained prospective results of applying the developed materials on cells and animals, further research needs to include an assessment of its safety and effectiveness of application in humans.

## Data Availability

Available upon request.
